# Genetic Variability and Population Structure of *Disanthus cercidifolius* subsp. *longipes* (Hamamelidaceae) Based on AFLP Analysis

**DOI:** 10.1371/journal.pone.0107769

**Published:** 2014-09-24

**Authors:** Yi Yu, Qiang Fan, Rujiang Shen, Wei Guo, Jianhua Jin, Dafang Cui, Wenbo Liao

**Affiliations:** 1 Guangdong Key Laboratory of Plant Resources and Key Laboratory of Biodiversity Dynamics and Conservation of Guangdong Higher Education Institutes, School of Life Sciences, Sun Yat-Sen University, Guangzhou, China; 2 College of Forestry, South China Agriculture University, Guangzhou, China; 3 Department of Horticulture and Landscape Architecture, Zhongkai University of Agriculture and Engineering, Guangzhou, China; Wuhan Botanical Garden, Chinese Academy of Sciences, Wuhan, China

## Abstract

*Disanthus cercidifolius* subsp. *longipes* is an endangered species in China. Genetic diversity and structure analysis of this species was investigated using amplified fragments length polymorphism (AFLP) fingerprinting. Nei's gene diversity ranged from 0.1290 to 0.1394. The AMOVA indicated that 75.06% of variation was distributed within populations, while the between-group component 5.04% was smaller than the between populations-within-group component 19.90%. Significant genetic differentiation was detected between populations. Genetic and geographical distances were not correlated. PCA and genetic structure analysis showed that populations from East China were together with those of the Nanling Range. These patterns of genetic diversity and levels of genetic variation may be the result of *D. c.* subsp. *longipes* restricted to several isolated habitats and “excess flowers production, but little fruit set”. It is necessary to protect all existing populations of *D. c.* subsp. *longipes* in order to preserve as much genetic variation as possible.

## Introduction

*Disanthus* Maxim. (Hamamelidaceae) is a monotypic genus endemic to China and Japan [1]. *Disanthus cercidifolius* subsp. *longipes* is the only subspecies in *Disanthus* which is distributed in China (southern Zhejiang, central and northwestern Jiangxi, and southern Hunan), while its sister, *D. c.* subsp. *cercidifolius*, is endemic to Japan [2]. *D. c.* subsp. *longipes* was first reported by Cheng [3] in 1938, and then revised by Chang [4] in 1948. In a systematic study of *Disanthus* [2], the author believed that the Chinese species of *Disanthus* was a sister population of *D. c.* subsp. *cercidifolius*, which is distributed in warm and humid forests of the Cathayan Land, and also was a Tertiary relic. *D. c.* subsp. *longipes* was characterized by its morphology and preference for humid, acid soils and shady habitats. The inflorescences are paired, axillary; Capitula are 2-flowers, purple; Leaves are heart shaped, green then turning to purple, orange, and red. It is usually a small tree, 2–3 m high, occasionally reaching heights of 6–8 m in forests when growing along streams. Because of severe habitat fragmentation that caused population decline, *Disanthus* was listed in the 1992 Red List of Endangered Plant Species of China [5], the 1994 IUCN Red List of Threatened Species (www.iucnredlist.org), the Key Wild Plants under State Protection [6] and the 2004 China Species Red List as bring a species at high risk of extinction in the wild [7].

Although study of *Disanthus* has attracted many investigators [8]–[22], little attention has been paid to its genetic analysis and population structure, except Xiao [23]. Xiao investigated the genetic diversity of *D. c.* subsp. *longipes* based on nine allozyme loci and found a higher genetic variation within populations as well as significantly lower variation among populations. However, sampling of Xiao's study was limited to part of the distribution area of *D. c.* subsp. *longipes* only. So a comprehensive study of the populations' genetic structure at different geographic scales is still needed.

Preserving the genetic diversity of endangered species is one of the primary goals in conservation planning. Because survival and evolution of species depended on the maintenance of sufficient genetic variability within and among populations to accommodate new selection pressures caused by environmental changes [24], [25]. For endemic endangered species, intraspecific variation is a prerequisite for any adaptive changes or evolution in the future, and have profound implications for species conservation [26], [27]. The knowledge of the levels and patterns of genetic diversity is important for designing conservation strategies for threatened and endangered species [28], [29]. So identifying variations with molecular markers has provided the abundant information concerning genetic diversity in plant species [30]–[36]. Amplified fragment length polymorphism (AFLP) [37] is a PCR-based technique which has been successfully applied to the identification and estimation of molecular genetic diversity and population structure [32]–[34], [38]–[41]. This technique can generate information on multiple loci in a single assay without prior sequence knowledge, and [42]. Using AFLP markers, we would know (1) the degree of genetic diversity within and among populations; (2) which factors might explain genetic variation; and (3) how to apply this information to develop recommendations for management of this endangered species.

## Materials and Methods

### Ethics Statement

Field studies were approved by Hunan Provincial Bureau of Forestry for collection in Xinning County (1XN), the Dupanglin National Nature Reserve (2DP), the Mangshan National Nature Reserve (3MS), and approved by Guangdong Provincial Bureau of Forestry for collection in Nanling National Nature Reserve (4NL), and approved by Zhejiang Provincial Bureau of Forestry for collection in QianJiang Source National Forestry Park (5QJ) and Zhulong town, Longquan City (6ZL), and approved by Jiangxi Provincial Bureau of Forestry for collection in Guanshan National Nature Reserve (7GS), and Mount Sanqingshan National Park (8SQ).

### Specimen collection

**Figure 1:**
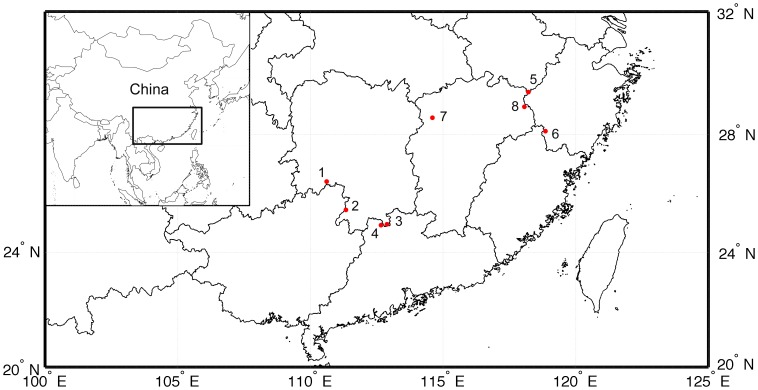
Location of eight populations in two groups sampled in a study of genetic diversity of *Disanthus cercidifolius* subsp. *longipes*. Populations are represented by black dots and located as Table 1. Note: 1:1XN, 2:2DP, 3:3MS, 4:4NL, 5:5QJ, 6:6ZL, 7:7GS, 8:8SQ.

The specimens were collected from eight populations from April to June of 2008. All populations but one grew in evergreen and deciduous broad-leaved mixed forests (often lived with species such as in genus *Cyclobalanopsis* and *Sorbus*, *etc.*) at altitudes of 450–1200 m. The exception (3MS) was the one growing in bamboo forests in the Mangshan Mountains of Yizhang. For molecular analysis, 10–11 individuals per population were sampled. The locations and information of populations are provide in Table 1 and Fig. 1. Before DNA extraction, all the dried leaves were preserved in silica gel [43]. All the voucher specimens are deposited at the Herbarium of Sun Yat-sen University (SYS).

**Table 1:** Information of location of populations.

ID	GPS co-ordinates	Location of populations	Location type	Altitude (m)	Collection voucher
1XN	26°25'08''N, 110°36'31''E	Xinning county, Hunan Province	mountain slope on the edge of forests	690–740	SHEN Rujiang and GUO Wei
2DP	25°27'43''N, 111°20'20''E	Dupanglin National Nature Reserve, Hunan Province	in bushes by stream	700–850	SHEN Rujiang and GUO Wei
3MS	24°58'12''N, 112°53'16''E	Mangshan National Nature Reserve, Hunan Province	in bamboo forests by stream	680–923	SHEN Rujiang and GUO Wei
4NL	24°56'18''N, 112°39'40''E	Nanling Naitional Nature Reserve, Guangdong Province	mountain slope	680–700	SHEN Rujiang
5QJ	29°23'59''N, 118°12'58''E	Qianjiang Source National Forestry Park, Zhejiang Province	mountain slope	650–700	SHEN Rujiang and GUO Wei
6ZL	28°06'28''N, 118°51'27''E	Zhulong town, Longquan City, Zhejiang Province	mountain slope by streams	1040	SHEN Rujiang and GUO Wei
7GS	28°33'22''N, 114°35'42''E	Guanshan National Nature Reserve, Jiangxi Province	mountain slope	560–600	CHEN Lin
8SQ	28°54'57''N, 118°03'52''E	Mount Sanqingshan National Park, Jiangxi Province	mountain slope in forests	620–1260	Observation team of Sun Yat-sen University

### DNA extraction and AFLP reactions

Genomic DNA was extracted from silica gel-dried leaves using the cetyl trimethylammonium bromide (CTAB) method [44]. The extracted DNA was dissolved in 100 µL of Tris-hydrochloride (TE) buffer [10 mmol/l Tris-HCl (pH 8.0), 1 mmol/EDTA (pH 8.0)] and used as a template for the polymerase chain reaction (PCR).

AFLP reactions were performed following the method reported by [37] with the following modifications. The restriction digest and ligation steps were done as separate reactions. For the digestion, approximately 500 ng of genomic DNA was incubated at 37°C (EcoRI) or 65°C (MseI) for 2 h in a 20 µL volume reaction containing 10× H Buffer (TOYOBO, Shanghai) and 10 U restriction enzymes EcoRI or MseI. For the ligation, 20 µL of a ligation mix consisting of 10× T4 DNA Ligase (TOYOBO, Shanghai), 1 µL EcoRI-adapter, 1 µL MseI-adapter, and 2 U T4 DNA Ligase was added to the sample and kept at 22°C for 3 h. After ligation, the samples were diluted 10-fold with sterile deionized water (dH_2_O). A pre-selective polymerase chain reaction (PCR), using PTC-200 thermocycler (MJ research, Waltham, MA) was done using primer pairs with a single selective nucleotide extension. The reaction mix (total volume 20 µL) consisted of 4 µl template DNA from the restriction/ligation step, 1 µL primer (EcoRI/MseI), and 15 µL AFLP Core Mix (13.8 µL dH_2_O, 1.6 µL MgCl_2_, 1.6 µL dNTPs (2.5 mM), 1 U Taq DNA polymerase, and 10× H buffer). After an initial incubation at 94°C for 2 min, 20 cycles at 94°C for 20 s, 56°C for 30 s, and 72°C for 2 min, with a final extension at 60°C for 30 min, were performed. The PCR products of the amplification reaction were diluted 10-fold with dH_2_O and used as a template for the selective amplification using two AFLP primers, each containing three selected nucleotides.

Nine primer combinations labeled with fluorescent 6-carboxy fluorescein (6-FAM) were probed for selective amplification, and only primer combinations with the greatest number of bands (EcoRI/MseI: AAC/CTG; ACA/CTG and ACT/CAT) were selected. Selective amplification was done using the following touchdown PCR conditions: 94°C for 2 min first, then 10 cycles at 94°C for 20 s, and 66°C for 30 s, with a 1°C decrease per cycle then extension at 72°C for 2 min; followed by 20 cycles at 94°C for 20 s, 56°C for 30 s, and 72°C for 2 min. After amplification, 3 µL of the samples were diluted 3-fold with sterile deionized water (dH_2_O), and mixed with 10 µL formamide and 0.2 µL Size standard-600 (Beckman Coulter, Fullerton, CA). The mix was used for sequence analysis.

Raw data were collected on a CEQ8000 Sequencer (Beckman Coulter). AFLP products were resolved using a Beckman Coulter CEQ8000 genetic analyzer. Semi-automated fragment analysis was performed using the fragment analysis software of the CEQ8000. The chromatograms of fragment peaks were scored as present (1) or absent (0), and a binary qualitative data matrix was constructed. A total of 82 individuals were run twice with all primer combinations.

### Data analysis

As a measure of population diversity, the binary data matrix was input to POPGENE version 1.32 [45], assuming Hardy–Weinberg equilibrium. The following indices were used to quantify the amount of genetic diversity within each population examined: the number of AFLP fragments (Frag_tot_), the percentage of polymorphic fragments (Frag_poly_), Nei's (1973) gene diversity (H), and Shannon's information index (I). In addition, the number of unique fragments (Frag_uni_) and DWs, frequency-down-weighted marker values [46], were calculated as measures of divergence. For each population, the number of occurrences of each AFLP marker in that population was divided by the number of occurrences of that particular marker in the total dataset, then these values were summed up [46], [47]. The value of DW was expected to be high in long-term isolated populations where rare markers should accumulate due to mutations, whereas newly established populations were expected to exhibit low values [46]. To even out the unequal sample sizes, DWs were calculated using five randomly chosen individuals. The value of DW was calculated by AFLPdat [48], which is based on R version 2.9.0 (www.r-project.org).

Nei's genetic distance [49] of *D. c.* subsp. *longipes* populations were calculated using the software POPGENE version 1.32 [45], then cluster analysis was performed based on the unweighted pair group method with arithmetic averaging (UPGMA) [50] using NTSYS pc version 2.1e [51]. In addition, based on the genetic distance index devised by [52], the UPGMA dendrogram of individuals was drawn using TREECON version 1.3b [53]. The robustness of the branches was estimated by 1000 bootstrap replicates.

ARLEQUIN version 3.0 [54] was used to perform an analysis of molecular variance (AMOVA) [55] to assess the hierarchical genetic structure among populations and within populations. The AFLPdat program [48], based on R version 2.9.0 (www.r-project.org), was used to convert the AFLP data matrix to the ARLEQUIN input format. The AMOVA was performed by partitioning genetic variation among and within populations regardless of their geographic distribution. In this study, the traditional *F*-statistics [56] cannot be used in dominant marker AFLP, therefore *Φ*-statistics [55] replace *F*-statistics. The significance of *Φ* values was tested by 1000 permutations. *Φ*-statistics are computed from a matrix of Euclidean squared distances between every pair of individuals [40]. Two models of the eight populations were tested to investigate regional relationships. Firstly, we treat all populations as a single group to obtain a value for *Φ*_st_ as an overall measure of population divergence (a two-level analysis), and then we divided the populations into two groups, the East China region and the Nanling Range (three-level hierarchical analyses).

A distance matrix of *Φ*_st_ between every pair of populations was calculated in ARLEQUIN as a measure of interpopulation genetic differentiation, from which 1000 bootstrapped replicate matrices were then computed, so gene flow (*Nm*) based on *Φ*_st_ [56] could be calculated. Isolation-by-distance was investigated by computing the correlation between geographic distance and genetic distance (*Φ*_st_) between every pair of populations and applying the Mantel test, using NTSYSpc version 2.1e [51]. The Mantel *z*-statistic value was tested non-parametrically by creating a null distribution of *z* using 1000 random permutations and comparing the observed *z* value [40].

The AFLP data were also subjected to a principal components analysis (PCA), which may help reveal unexpected relationships among a large number of variables, reducing them to two or three new uncorrelated variables so they retain most of the original information [57]. We chose Jaccard's similarity coefficient [50] to calculate the eigenvalue and eigenvector. The standardized data were projected onto the eigenvectors of the correlation matrix and represented in a two-dimensional scatter plot [57]. Plots of samples in relation to the first three principal components were constructed with populations designated as either populations of the East China region or populations of the Nanling Range. The data from the PCA was analyzed using the computer program NTSYSpc version 2.1e [51]. A two-dimensional representation of genetic relationships among *D. c.* subsp. *longipes* genotypes was carried out using SPSS version 16.0 [58].

STRUCTURE version 2.3.1 [59] was used to investigate structure at the individual level. In this study, we inferred that each individual of *D. c.* subsp. *longipes* comes purely from one of the populations. It was applied using a “no admixture” model, 100 000 burn-in period, and 50 000 MCMC replicates after burn-in. The approach requires that the number of clusters K be predefined, and the analysis then assigns the individuals to clusters probabilistically [40]. We performed ten runs for each value of K (1 to 10). To determine the K value, we used both the LnP(D) value and Evanno's ΔK [60]. LnP(D) is the log likelihood of the observed genotype distribution in K clusters and can be output by STRUCTURE simulation [59]. Evanno's ΔK took consideration of the variance of LnP(D) among repeated runs and usually can indicate the ideal K. The suggested Δk  =  M(|L(k+1)-2L(k) +L(k-1)|)/S[L(k)], where L(k) represents the kth LnP(D), M is the mean of 10 runs, and S their standard deviation[60], [61]. The output uses color coding to show the assignments of individuals in each population to the clusters.

Population structure was also investigated by HICKORY version 1.1 [62], in order to assess the importance of inbreeding in the data and the assumption of Hardy-Weinberg equilibrium. HICKORY makes it possible to evaluate departures from Hardy-Weinberg equilibrium in dominant as well as co-dominant markers [40]. The program AFLPdat [48] was used to convert the AFLP data matrix to the HICKORY input format. The deviance information criterion (*DIC*) criteria, *Dbar*, *Dhat*, and *pD*, for assessing the importance of inbreeding were computed using the default values: Burn-in 5000; sample 100 000; thin 20 [62].

## Results

### Population genetic diversity

In this study, three of the nine AFLP primer combinations were used (Table 2). The number of fragments for each primer combination (with percentage of polymorphisms within parenthesis), were: EcoRI-AAC/MseI-CTG: 144 (98.61%), EcoRI-ACA/MseI-CTG: 152 (99.34%), and EcoRI-ACT/MseI-CAT: 157 (96.18%). The length of the fragments varied from 64 bp to 560 bp. The three AFLP primer combinations produced a total of 453 fragments in 82 individuals, of which 444 (98.01%) were polymorphic. The total number of fragments per population (Frag_tot_) varied between 198 (7GS) and 227 (5QJ). The percentage of polymorphism (Frag_poly_) across the eight populations ranged from 43.71% (7GS) to 50.11% (5QJ). The number of fragments that only occur in one population (Frag_uni_) varied between 6 (7GS) and 16 (8SQ). The DW ranged from 56.85 (5QJ) to 111.19 (1XN), mean 76.88, and SD  = 21.11. The diversity within a population (H) ranged from 0.1290 (2DP) to 0.1394 (5QJ) (Table 3).

**Table 2:** Number of loci evaluated for each of three AFLP primer combinations utilized in assays of 82 individuals of *Disanthus cercidifolius* subsp. *longipes* from eight populations.

Primer combination	Number of loci
EcoRI-AAC/MseI-CTG	144
EcoRI-ACA/MseI-CTG	152
EcoRI-ACT/MseI-CAT	157
Total	453

**Table 3:** Region, population (ID), sample size (N), total number of fragments per population (Frag_tot_), percentage of polymorphic fragments (Frag_poly_), number of fragments that only occur in one population (Frag_uni_), frequency-down-weighted marker values (DW), Nei's (1973) gene diversity (H), Sannon's index (I) sampled in a study of *Disanthus cercidifolius* subsp. *longipes* (H. T. Chang) K. Y. Pan populations.

Region	ID*	N	Frag_tot_	Frag_poly_	Frag_uni_	DW	H	I
Nanling range	1XN	10	216	47.68	12	111.19	0.1357±0.1779	0.2106±0.2570
	2DP	11	203	44.81	11	56.96	0.1290±0.1790	0.1991±0.2580
	3MS	10	204	45.03	10	93.96	0.1373±0.1839	0.2100±0.2652
	4NL	10	215	47.46	9	58.09	0.1383±0.1710	0.2136±0.2608
East China region	5QJ	11	227	50.11	10	56.85	0.1394±0.1778	0.2172±0.2564
	6ZL	10	209	46.14	12	77.45	0.1393±0.1822	0.2141±0.2631
	7GS	10	198	43.71	6	64.85	0.1353±0.1852	0.2062±0.2662
	8SQ	10	223	49.23	16	95.68	0.1373±0.1775	0.2138±0.2560
All	-	82	453	98.01	-	-	0.1781±0.1633	0.2918±0.2223

### AMOVA

The two-level AMOVA in ARLEQUIN gave a *Φ*_st_ value of 0.2328 (P<0.001), with of 23.28% variation among populations and 76.72% within populations (Table 4). The three-level hierarchical AMOVA analyses of the two-group models shows that three-quarters of the variation (75.06%) was concentrated within populations, while the between group component (5.04%) was less than the between populations-within-group component (19.90%). All three *Φ* values were significant based on a 1000 permutation test (Table 4).

**Table 4:** Analysis of molecular variance (AMOVA) in *Disanthus cercidifolius* subsp. *longipes* for 82 individuals from eight populations.

Model	Source of variation	df	Sum of squares	Variance component	Percentage variance %	*Φ*-statistics	*P*
Two levels	among populations	7	1055.95	11.14	23.28	*Φ*_st_ = 0.23283	*P*<0.001
	within populations	74	2716.05	36.7	76.72		
	Total	81	3772	47.84			
three levels	between groups	1	237.66	2.46	5.04	*Φ*_ct_ = 0.05040	*P*<0.05
	between populations-within-groups	6	818.29	9.73	19.90	*Φ*_sc_ = 0.20955	*P*<0.001
	within populations	74	2716.05	36.70	75.06	*Φ*_st_ = 0.24939	P<0.001
	Total	81	3772	48.89			

Computed with ARLEQUIN version 3.0. The *P* values represent the probability of obtaining an equal or more extreme value by chance, estimated from 1000 permutations.

### Pairwise genetic distance and gene flow

The pairwise genetic distance (*Φst*) and gene flow (*Nm*) matrix were used to establish the level of genetic divergence among the populations (Table 5). Estimates of pairwise genetic distance using AFLP date ranged from 0.1364 (*P*<0.001) for the most closely related populations (4NL and 6ZL), to 0.3428 (*P*<0.001) in the most divergent populations (3MS and 8SQ). The gene flow (*Nm*) among populations ranged from 0.4792 to 1.5830. Except for one population in Nanling National Nature Reserve (4NL), most of populations' *Φst* were above 0.2, and the corresponding *Nm* were below 1. These results indicate that the genetic distance of *D. c.* subsp. *longipes* is not close, yet nor dependent on geographic distance.

**Table 5:** Pairwise genetic distance (*Φst*, lower diagonal, *P*<0.001) and gene flow (*Nm*, upper diagonal) between eight populations of *Disanthus cercidifolius* subsp. *longipes* based on AFLP data.

	1XN	2DP	3MS	4NL	5QJ	6ZL	7GS	8SQ
1XN	-	1.4673	0.7122	1.1283	0.7637	0.7610	0.8506	0.6140
2DP	0.1456	-	0.8055	1.1586	0.6833	0.7813	0.7398	0.5624
3MS	0.2598	0.2369	-	1.4639	0.6874	0.7389	0.8037	0.4792
4NL	0.1814	0.1775	0.1459	-	1.0727	1.5830	1.2885	0.6644
5QJ	0.2466	0.2679	0.2667	0.1890	-	1.1441	0.7897	1.3148
6ZL	0.2473	0.2424	0.2528	0.1364	0.1793	-	1.1783	0.6917
7GS	0.2272	0.2526	0.2373	0.1625	0.2405	0.1750	-	0.5466
8SQ	0.2893	0.3077	0.3428	0.2734	0.1598	0.2655	0.3138	-

Significance levels are based on 1000 iterations and indicate that the probability that random *Φst* values are higher than the observed values. *Nm*  =  (1 - *Fst*)/4*Fst [56].

### Relationship between geographic distance and genetic distance (The Mantel tests)

**Figure 2:**
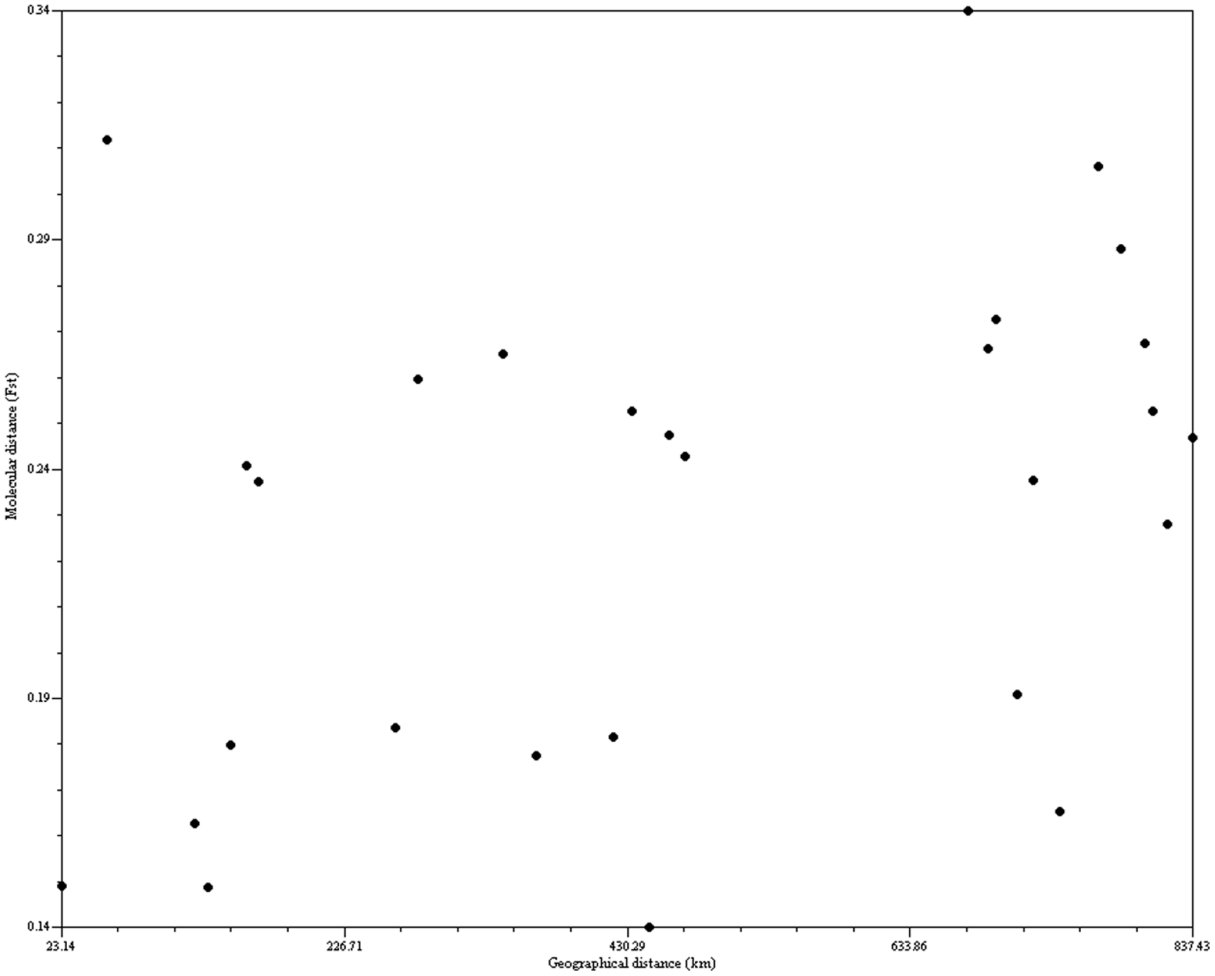
*Disanthus cercidifolius* subsp. *longipes*, bivariate plot showing no correlation between matrices of pair-wise geographic (km) and genetic distance (*Φst*) in eight populations, comprising 82 individuals. Computed with NTSYSpc ver. 2.1e.

A Mantel test showed no correlation between the geographic distance and the *Φst* genetic distance (r = 0.38917, *P* = 0.9920, Fig. 2). This result implies that *D. c.* subsp. *longipes* does not demonstrate a historical pattern of isolation-by-distance.

### Principal components analysis

**Figure 3:**
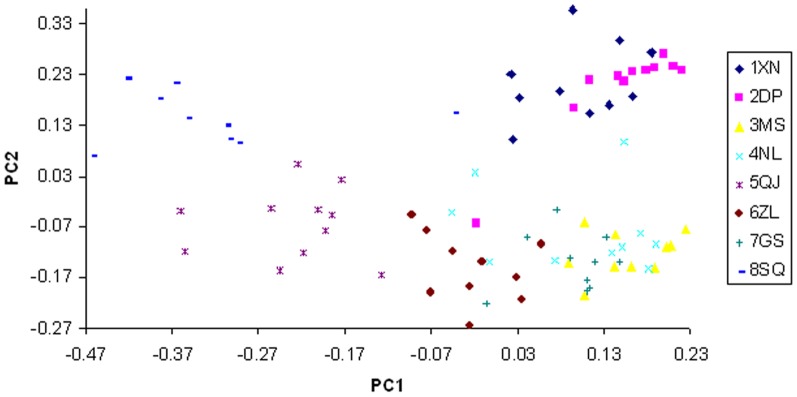
Principal coordinates analysis of 82 individuals from eight populations, based on dissimilarity matrix (Jaccard's coefficient). Accessions are plotted according to the values of first (x axis) and the second (y axis) components and with different symbols according to population. Principal coordinate axes shown (pc1 and pc2) represent 25.46% and 25.17% of respective variance in the dissimilarity matrix.

A PCA of the AFLP-based distance data was performed to examine relationships among the populations of *D. c.* subsp. *longipes*. The PCA showed that populations from East China and the Nanling Range clearly do not cluster separately (Fig. 3). The natural divide is separated into three clusters: cluster 1 (1XN, 2DP), cluster 2 (5QJ, 8SQ), and cluster 3, a mixture that includes some individuals from cluster 1 or cluster 2. The first and second principal component axes, PC1 and PC2, accounted for 25.46% and 25.17% of the total variation, respectively.

### Hierarchical and cluster analysis

**Figure 4:**
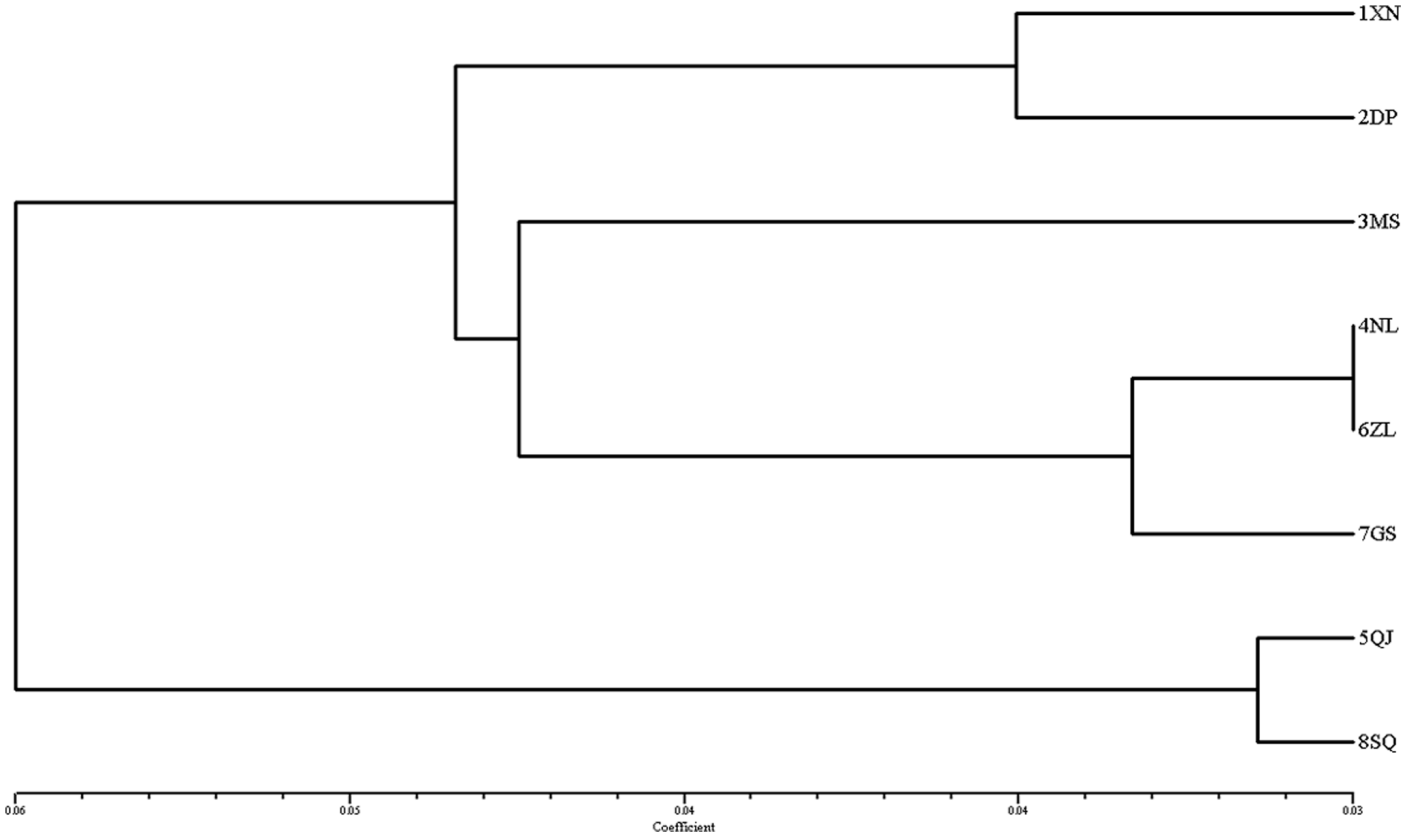
UPGMA dendrogram based on genetic distance. Note: A = 1XN, B = 2DP, E = 3MS, G = 4NL, H = 5QJ, I = 6ZL, J = 7GS, K = 8SQ.

**Figure 5:**
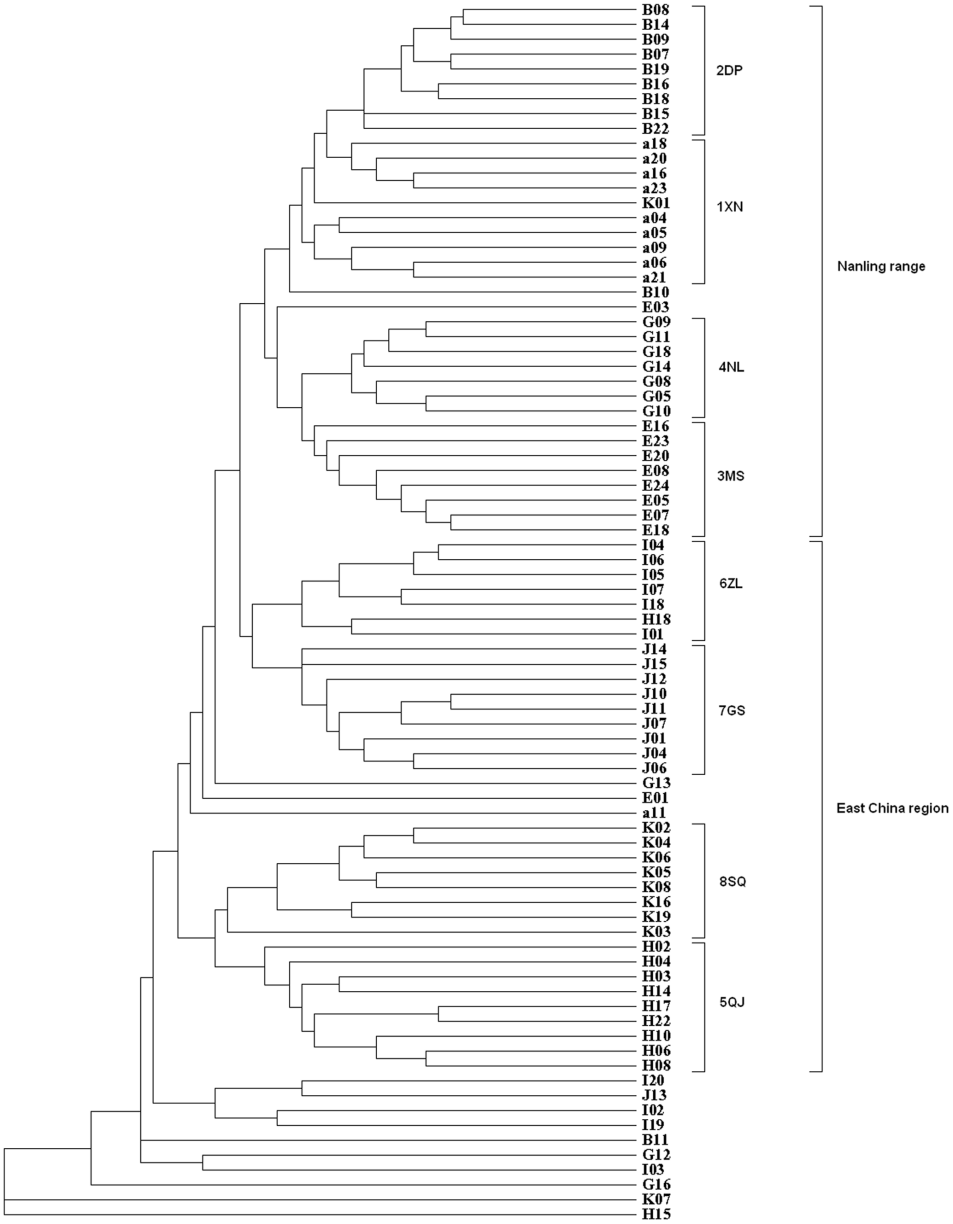
The UPGMA dendrogram based on Nei & Li's genetic distance.

Nei's genetic distances [49] between populations showed two main clusters of populations (Fig. 4). The cluster that comprises 5QJ and 8SQ is clearly differentiated from the others. Populations 1XN and 2DP are another cluster, and population 4NL and 6ZL are grouped with 7GS, although the UPGMA dendrogram of the 82 individuals based on Nei and Li's genetic distance is not clearly grouped (Fig. 5). Although most individuals in the same population cluster together, some individuals in different populations (except 1XN and 3MS) also are clustered together.

**Figure 6:**
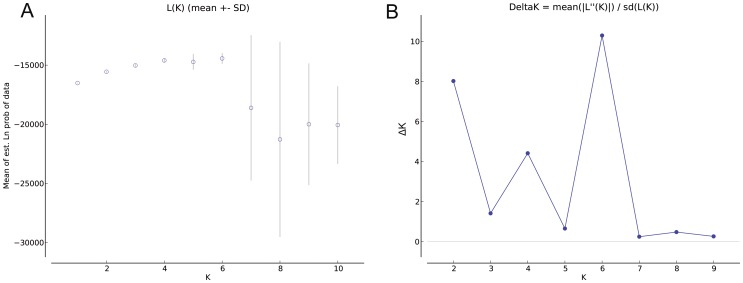
The mean LnP(D) and ΔK over 10 repeats of STRUCTRUE simulations. A. Mean lnP(D) value with k = 1–10. B. ΔK value with k = 2–9.

**Figure 7:**
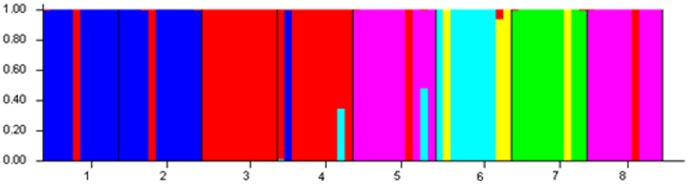
Diagram of results from STRUCTURE(k = 6).

In the genetic structure analysis (STRUCTURE), the highest estimate of the likelihood of the data, given the number of clusters chosen, was k = 10 (ten clusters). We can't get a clear knee in Lnp(D) (Fig. 6). Evanno's ΔK took consideration of the variance of LnP(D) among repeated runs and usually can indicate the best k. See the Fig. 6, we can find that when the k = 6 and we get the highestΔK value. So the best k = 6. The diagram (Fig. 7) showing assignment of individuals to the clusters revealed a clinal structure to the data. In the six-cluster model (k = 6), gene pool 1 (including 5QJ, 8SQ) and gene pool 2 (including 1XN, 2DP) are restricted with a few from another pool, only being distributed in the southwest (gene pool 1) and northeast (gene pool 2). Gene pool 3 (including 3MS, 4NL), while Gene pool 4, pool 5 and pool 6, consist of 6ZL and 7GS. These 4 pools have the most widespread pattern.

The HICKORY results showed that there is inbreeding in populations of *D. c.* subsp. *longipes* (Table 6), because the *DIC* and *Dbar* parameter were lower in “*Full model*” than in other models. This pattern of results allows the “*Full model*” to be considered best, according to the HICKORY manual [62].

**Table 6:** Genetic structure analysis using HICKORY ver. 1.1 [62].

	Parameter				
Model	*Dbar*	*Dhat*	*pD*	*DIC*	*f*
*Full*	6750.93	5395.03	1355.9	8106.82	0.980572
*f = 0*	6780.4	5350.93	1429.47	8209.87	
*Theta = 0*	12092.5	11716.4	376.128	12468.6	0.994715
*f free*	6844.98	5363.43	1481.55	8326.53	0.500214

Default values for computations were used as follows: Burn-in 5000; sample 100 000; thin 20.

## Discussion

### Genetic diversity in endemic and endangered species

Accurate estimates of genetic diversity are useful for optimizing sampling strategies aiming at the conservation and management of genetic resources [26], [63], [64]. According to Hamrick and Godt (1989), there are strong associations between geographic range and genetic diversity [65]. Allozyme analyses concluded that endemic and geographically limited plant species generally possess less genetic variation, due to genetic drift and restricted gene flow [66]–[68]. However, in our study, the percentage of polymorphic fragments (Frag_poly_) and Shannon's information index (I) are higher than those of endemic species based on allozyme [65]. This may account for the new technique can generate more genetic diversity information. Historical events have also been shown to be responsible for variations in genetic diversity [68]. Numerous allozyme studies and an increasing number of cpDNA and mtDNA studies now provide substantial evidence that putative refugee plant populations harbor higher levels of genetic diversity relative to their likely descendant populations [69]. *Disanthus cercidifolius* subsp. *longipes* is a Tertiary relic, which is distributed in warm and humid forests of the Cathayan Land in the Tertiary [2], [70]. Lots of present endemics, several of which inhabit Pleistocene refugia during the Quaternary glacial period, were able to maintain higher levels of diversity because of population stability during the glacial cycle. *Disanthus cercidifolius* subsp. *longipes* may be one of them and preserved mutation during the relatively long glacial period. So it shows high genetic diversity [69], [71]. Compared by AFLP, RAPD, and ISSR markers, the genetic diversity indices in this study revealed an intermediate level of genetic diversity of *D. c.* subsp. *longipes* compared to other endangered species [34], [41], [66], [72], [73].

Results of studies of genetic diversity within populations show that all populations were on the similar level, except for the Dupanglin National Nature Reserve (2DP) which is shown to be lower than the others. The lack of genetic diversity resulting from the total existing individual in population 2DP is limited (fewer than 100 individuals). In practice, a larger population often has higher genetic variation [72], [74].

### Genetic variation and gene flow

In this study AMOVA showed that the largest portion (76.72%) of genetic variance is contributed by genetic variation within populations (*Φ_st_* value 0.23283) and only a small portion (23.28%) is due to differences among groups. Some other studies of endemic and endangered species show a similar pattern [34], [41], [66], [72], [73]. Moderate or high diversity and low population partitioning in rare plants have previously been attributed to a number of factors [66], [75]–[77], including insufficient length of time for genetic diversity to be reduced following a natural reduction in population size and isolation; adaptation of the genetic system to small population conditions; recent fragmentation (human disturbance) of a once-continuous range, i.e., genetic system; and extensive gene flow due to the combination of bird pollination and high outcrossing rates [66], [78]. The direct estimate of gene flow (*Nm*) based on *Φ_st_* 0.23283 was 0.82374, which means that the number of migrants per generation is lower than one. The present distribution of *D. c.* subsp. *longipes* is restricted to several isolated habitats [5], [7]. However, fossil and palynological evidences suggested that, *Disanthus* was wide spread in warm and humid forests in the Tertiary [2], [70]. Accordingly, a reasonable hypothesis is that the modern range of *Disanthus* species was the result of population fragmentation and contraction after the Quaternary glacial cycles. Considering that *D. c.* subsp. *longipes* has poor success in pollination, “excess flowers production, but little fruit set” [79], and fragmented habitat, inbreeding, which generally leads to decreased fitness, or inbreeding depression, of a population, some of the above causes result in the endangered status of *D. c.* subsp. *longipes*.

### Genetic structure at different hierarchical levels

Although AFLP markers are dominant, they provide no information on heterozygote frequencies, and our investigation provides no direct information on the reproductive strategy of *D. c.* subsp. *longipes*. In general, outcrossing and long-lived seed plants maintain the most genetic variation within populations, while predominantly selfing, short-lived species harbor comparatively higher variation among populations [65], [80]. According to data based primarily on allozyme analysis, *Φ_st_*  = 0.2 for outcrossing species and 0.5 for inbreeders [65]. The overall *Φ_st_* value 0.23283 and the gene flow value for *D. c.* subsp. *longipes* are similar to other species that outcross [81]–[84]. Permutation tests of the fixation index (*Φ_st_*  = 0.23) indicated significant genetic structuring. The populations of *D. c.* subsp. *longipes* spread from east to west (Fig. 1). And in the field investigation of our study, we found that *D. c.* subsp. *longipes* is one of the dominant species in its habitat.

HICKORY software analysis suggests that *D. c.* subsp. *longipes* populations are not genetically differentiated. There is inbreeding in populations of *D. c.* subsp. *longipes* (Table 6), because the *DIC* and *Dbar* parameter were lower in “*Full model*” than that in other models [62]. While inbreeding generally leads to decreased fitness (inbreeding depression) of a population, it also can be advantageous, allowing plants to adapt to disadvantageous conditions [85]–[88]. *D. c.* subsp. *longipes* originally had an outcrossing system, but under some conditions (e.g., poor efficiency in wind or insect pollination), it would adopt inbreeding or mixed systems to reduce the risk of reproductive failure. This result is consistent with Xiao's conclusions [89] from his study of the reproductive ecology of *D. c.* subsp. *longipes*. If we use co-dominant markers, it most likely would provide an affirmative result and better understanding of the processes.

Hierarchical cluster analysis (Fig. 5) revealed that most individuals of a specific population were grouped together, despite the mixing-in of some individuals from different populations. Most geographically close populations tended to cluster together. The DW values show that the two close populations are clearly divergent, that one is long-term isolated (DW value higher) and the other is newly established (DW value lower). The DW value of 7GS is close to average, but its site is isolated. It might be that there once had been other populations between 6ZL and 7GS. In fact, there is a population of *D. c.* subsp. *longipes* in the Junfeng Mountains, Jiangxi Province, according to Pan's data [2], but we have not obtained any specimens for analysis since 2008. Moreover, although *D. c.* subsp. *longipes* in the Jinggang Mountains, Jiangxi Province, might have been transplanted from Guanshan National Nature Reserve based on the data [90], we found a large population area of *D. c.* subsp. *longipes* in the Jinggang Mountains in October 2009, evidence that the present distribution of *D. c.* subsp. *longipes* appears to be relic from a once extensive range and population.

Genetic structure analysis of the individual samples using STRUCTURE shows that six gene pools are represented in the data, and each gene pool is relatively independent except a few mixtures from other clusters. 1XN and 2DP are the first cluster separated from the other populations; 5QJ and 8SQ form the distinct cluster 2 from the others; 3MS and 4NL also cluster with each other and become distinct from rest populations. These three clusters show high correlation with geographic distribution. And the rest three clusters mixed in 6ZL and 7GS. There is a close relationship between 6ZL and 7GS. It shows that 6ZL is related to 7GS rather than to 5QJ or 8SQ, although 6ZL is close to 5QJ and 8SQ in geographic location. It seems likely that there were some populations between current populations 6ZL and 7GS, or gene flow could have been accomplished by pollen or seed dispersal. The results of PCA based on AFLP data indicate a different result, which the eight populations form a triangle that has 1XN and 2DP at one angle, 5QJ and 8SQ at a second angle, but 3MS and 4NL mixed with 6ZL and 7GS in the third angle. The reasons for divergence of the East China region and the Nanling Range are not clear.

### Guide for conservation measures

In the face of the species extinction generated by human beings [91], [92], the urgent need for the conservation of biodiversity is global. In fact, many endemic species became endangered by the loss or fragmentation of habitats and change in natural conditions [26], [29], [64], [93], [94]. *D. c.* subsp. *longipes*, with its narrow archipelago-like distribution, habitat fragmentation, and reproductive-physiologic barriers, is typical species that can be easily affected. Although *D. c.* subsp. *longipes* is endangered in China, it has not yet been given high priority for protection. The original aim of this work is to provide some insight into the population biology of *D. c.* subsp. *longipes* in order to guide conservation measures. The guides which can be suggested by our study listed below. Firstly, Most of the genetic variation is located within populations, so we need to protect all the existing populations of *D. c.* subsp. *longipes* in order to preserve as much genetic variation as possible. Secondly, we should promote the further research and technology development to enable better reproduction of *Disanthus*. Finally, it is necessary to enforce the governmental law to forbid people stealing or purchasing wild *Disanthus*.

## Conclusions

The results of the AFLP on populations of *Disanthus cercidifolius* subsp. *longipes* show a pattern of high within-population diversity and low among-population divergence. Although the distribution of *D. c.* subsp. *longipes* could be grouped as the East China region and the Nanling Range, the population divergence is clear. The estimates of genetic differentiation and gene flow suggest that the species primarily outcrosses, but can resort to mixed reproductive strategies. These patterns of genetic diversity and levels of genetic variation may be the result of *D. c.* subsp. *longipes* restricted to several isolated habitats and “excess flowers production, but little fruit set”. The status of relic and their low reproductive success results in *D. c.* subsp. *longipes* being endangered.
